# Impaired Functional Connectivity in the Prefrontal Cortex: A Mechanism for Chronic Stress-Induced Neuropsychiatric Disorders

**DOI:** 10.1155/2016/7539065

**Published:** 2016-01-19

**Authors:** Ignacio Negrón-Oyarzo, Francisco Aboitiz, Pablo Fuentealba

**Affiliations:** Departamento de Psiquiatría, Facultad de Medicina, Centro Interdisciplinario de Neurociencia, Pontificia Universidad Católica de Chile, Avenida Marcoleta No. 391, 8320000 Santiago, Chile

## Abstract

Chronic stress-related psychiatric diseases, such as major depression, posttraumatic stress disorder, and schizophrenia, are characterized by a maladaptive organization of behavioral responses that strongly affect the well-being of patients. Current evidence suggests that a functional impairment of the prefrontal cortex (PFC) is implicated in the pathophysiology of these diseases. Therefore, chronic stress may impair PFC functions required for the adaptive orchestration of behavioral responses. In the present review, we integrate evidence obtained from cognitive neuroscience with neurophysiological research with animal models, to put forward a hypothesis that addresses stress-induced behavioral dysfunctions observed in stress-related neuropsychiatric disorders. We propose that chronic stress impairs mechanisms involved in neuronal functional connectivity in the PFC that are required for the formation of adaptive representations for the execution of adaptive behavioral responses. These considerations could be particularly relevant for understanding the pathophysiology of chronic stress-related neuropsychiatric disorders.

## 1. Introduction

The enriched image we have of the world as well as the great number of behavioral options implies the need for appropriate and adaptive accommodation of behavioral responses for our survival. This demand is especially evident in neuropsychiatric disorders like major depressive disorder (MDD), anxiety disorders (including posttraumatic stress disorder, PTSD), and schizophrenia (SZ), in which subjects develop aberrant and maladaptive behavioral responses which negatively affects well-being (reviewed in [1, 2]). For example, MDD is characterized by a bias in explicit memory that favors negative self-related information and increased bias to interpret perceptual stimuli as negative [3, 4]. Similarly, anxiety disorders are characterized by selective attention favoring threatening information and increased retention of aversive memories [3, 5, 6], while SZ patients display a tendency for ambiguous stimuli to be misinterpreted as threatening, together with misinterpretation of the intentions of others [2, 7]. Simultaneously, these disorders are also characterized by impaired behavioral flexibility, that is, the ability to adapt behavioral responses to current environmental demands [8]. This evidence suggests a clear impairment in the adaptive control of behavior.

The adaptive organization of goal-directed behavioral responses is supported by the prefrontal cortex (PFC) [9, 10] and its appropriate connectivity with other brain structures, as the hippocampus (HPC) and the amygdala [11–14]. It is argued that neuropsychiatric disorders involve alterations to the behavioral control system supported by the PFC and its connectivity with other brain systems [2, 15, 16]. In support of this idea, it has been shown that patients suffering from these disorders display alterations of PFC-dependent cognitive functions, like cognitive flexibility [17], working memory [18, 19], and fear extinction [20]. In the same line of evidence, functional alterations of the PFC have been observed in patients suffering from neuropsychiatric disorders [4, 21–24]. Also, these patients display altered functional connectivity in the PFC-HPC and the PFC-amygdala pathway [25–27]. These evidences suggest that such disorders are related to the maladaptive accommodation of behavioral responses supported by the PFC, and its connection with other structures. On the other hand, chronic stress, that is, an intense and sustained maladaptive response to environmental threats [28, 29], is considered one of the most important risk conditions for the development of neuropsychiatric disorders [30–32]. Therefore, connecting this evidence, it has been suggested that chronic stress affects the accurate functioning of the PFC [33, 34], which is expressed as the maladaptive generation of behavioral responses observed in neuropsychiatric disorders. However, how chronic stress affects the neural representation of behavior in the PFC and how these alterations are expressed as maladaptive behavioral responses remain still elusive.

In the present review we integrate theoretical considerations and empirical evidence, mainly from rodents animal models, to relate the effects of chronic stress on neural functional connectivity related to the organization of behavior mediated by the PFC. In Section 2, we approach theoretical frameworks proposing that the control of behavior is mediated by the formation of cognitive maps in the PFC. In Section 3, we propose these cognitive maps are represented as functional connectivity of neuronal assemblies in the PFC. We then in Section 4 propose synaptic plasticity and oscillatory synchrony as neurophysiological mechanisms involved in the formation of these neural assemblies in the PFC. We also report evidence of the chronic stress-induced impairment of these mechanisms. In Section 5, we approach the long-term storage of neural assemblies representing cognitive maps in the PFC, the neurophysiological mechanisms involved, and in Section 6 the consequences of chronic stress on these mechanisms. In Section 7, we propose a hypothesis for chronic stress-induced psychiatric disorders based on functional connectivity in the PFC. Finally, we discuss future research in this area, in particular concerning the pathophysiology of chronic stress-related neuropsychiatric disorders.

## 2. Adaptive Control of Behavior by the PFC

### 2.1. Function of the PFC

The first clues about the role of the PFC came from studies of PFC lesions in humans. Lesions of the PFC have long been known to produce impairment of cognitive functions, such as attention, set-shifting, working memory, planning, temporal integration, decision making, retrieving and manipulating old memories, and inhibitory control (reviewed extensively in [35]). Although rodents do not possess anatomical features of primate PFC, a large body of evidence has shown that lesions to the rodent medial-PFC (mPFC) subserves a range of cognitive and behavioral processes homologous to those mediated by the primate PFC (reviewed in [36], [37]). Lesions to the rodent mPFC impair set-shifting [38], working memory [39], and the recall of extinction of conditioned fear, a type of emotional reversal learning [40]. Thus, rodent models have important properties to the study of behavioral and neural properties related to the control of behavior.

The lesion studies of the PFC in both primates and rodents led to the idea that the principle and more general behavioral impairments associated with PFC lesions are not explained by failure of a particular function but imply a failure to coordinate a set of cognitive processes. A large body of evidence leads to the hypothesis that the main function of the PFC is cognitive control of behavior, that is, the adaptive accommodation of behavioral responses to current perceptual conditions toward the attainment of goals [41], also known as executive control [10]. To accomplish this function, the PFC integrates perceptual information about the current context and execute an appropriate behavioral response a process known as the perception-action cycle [9].

To the execution of the perception-action cycle, both the primate PFC and rodent mPFC are anatomically positioned between the perceptual input and motor and visceral output neural systems [9]. The perceptual information communicates about the internal state (needs), the significance and motivational and emotional information (valence and arousal), contextual information of the stimuli, and previous memories related to similar perceptual information.

Perceptual information about the external and internal environment is transmitted mainly from the orbitofrontal cortex (OFC) and the insula, respectively [42]. The PFC also receives inputs from the thalamus (mediodorsal and reuniens nuclei) [43], which is involved in learning new information, from the hippocampus (HPC), which is involved in spatial and episodic memory and in memory consolidation [44, 45], and from the amygdala, which carries information about the motivational significance of sensory stimuli [46, 47]. The ascending arousal system also projects densely to the PFC [48–51]. Thus, the PFC integrates perceptual, contextual, and motivational information about the environment, together with internal states and needs.

For the execution of the perception-action cycle, the PFC projects to neural systems involved in generating behavioral responses and executing actions [52, 53]. The PFC projects to several outputs, such as the premotor cortex, the hypothalamus (reviewed in [53]), the striatum [54], and the ascending-arousal system [55]. Thus, the PFC projects to motor, neuromodulatory, and visceral brain systems to generate an appropriate response according to internal and external demand in a goal-directed manner.

It is important to take into consideration that, based on anatomical and physiological criteria, both the primate and rodent PFC may be subdivided into different compartments which could have different behavioral roles (reviewed in [9, 52, 53]). Thus, for example, the rodent PFC is subdivided into the dorsal anterior cingulate cortex (Acc), the prelimbic cortex (PL), and infralimbic cortex (IL). Projection of the PL includes the agranular insular cortex, the claustrum, ACC (and extended ventral striatum), basolateral amygdala, the paraventricular, RE and MD of thalamus, VTA/SNc, and raphe nuclei of the midbrain (SLN, DR, and MR). Thus, the connectivity pattern of the dorsal mPFC is consistent with a role in limbic-cognitive functions homologous to the dorsolateral prefrontal cortex of primates. On the other hand, the IL projects mainly to forebrain and brainstem sites controlling autonomic/visceromotor activity, projections that are consistent with a role for IL in the control of visceral/autonomic activity homologous to the orbitomedial prefrontal cortex of primates [52, 53, 56]. Also PL and IL are strongly interconnected [53], suggesting the integration of both visceral and cognitive elements, which seems to be necessary to the generation of adaptive behavioral responses.

### 2.2. Formation of the Cognitive Map Related to Control of Behavioral in the PFC

**Figure 1:**
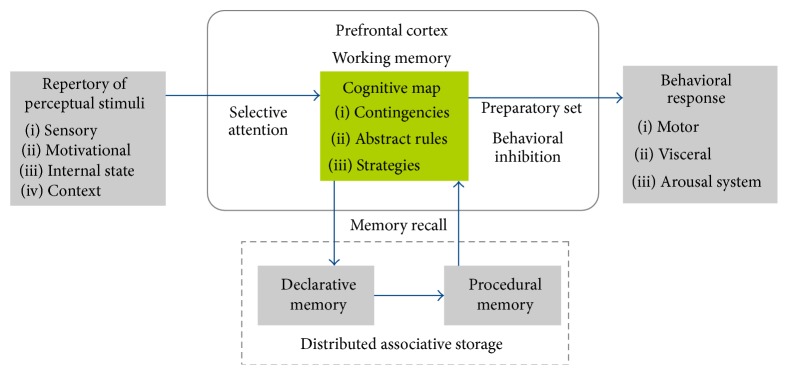
Cognitive map of the perception-action cycle in the PFC. We present a diagram of the execution of cognitive behavioral control by the PFC in the perception-action cycle. Sensory, motivational, contextual, and internal state information is sent to the PFC, where it is integrated through synaptic inputs from other brain systems (OFC, HPC, amygdala, insula, ascending arousal system, etc.). Cognitive function, as selective attention, participates in the integration of perceptual stimuli. Once integrated, a labile cognitive map is formed in the PFC, which represents the coupling of perception and actions as required for adaptive goal-directed behaviors. This link is provided by the projection of the PFC to neural systems involved in executing actions and behavioral responses (premotor cortex, striatum, ascending-arousal system, and hypothalamus). The cognitive map formed in the PFC contains contingencies, abstract rules, and strategies related to the accomplishment of the goal. Simultaneously, through memory recall, this cognitive map retains links to and among distributed associative memories in other brain systems (declarative and procedural memories), allowing for retrieving memories and other representations when needed. Cognitive function as working memory is involved in the “online” attainment of the cognitive map, while preparatory set and inhibitory control participates in the generation of accurate behavioral responses.

Theoretical work by Fuster [9, 57, 58] proposes that once perceptual information is selected and integrated in the PFC, a cognitive map of the perception-action cycle is formed in this structure that integrates and engages the repertory of perceptions and actions coupled as required in a specific adaptive goal-directed behavioral response [9, 58]. This cognitive map may include simple associations between cues and goals (contingency), and associations between different rules and strategies to accomplish goals (Figure 1).

In a first step, through selective attention, the perceptual information relevant to the goal is focused and selected. During the acquisition of the perception-action cycle, the PFC recalls and engages sparse representations of perception and of actions stored in different brain systems (declarative and procedural memories, Figure 1), allowing for memories and other tasks to be brought “online” when needed, for which executive functions like working memory are implemented. Other executive functions are also required, as a preparatory set for the selection of the action coupled with perceptual information, and inhibitory control to suppress cognitive or emotional contents and operations that may interfere with the goal.

After acquisition, the cognitive map may be labile in time, confirming the short-term nature of the perception-action cycle [56]. If the cognitive map has motivational significance, it is transformed into a static cognitive map in the PFC, corresponding to the remote memory of the perception-action cycle ([59] see below). If current perceptual information is similar to a previously stored cognitive map, it induces reactivation of the map (memory recall), a process that brings “to mind” the previously linked distributed associative representations through working memory as required [56]. Thus, stored cognitive maps are activated whenever the subject faces a similar environmental challenge. However, if perceptual stimuli are novel or ambiguous, the perceptual information is not explicitly linked to a clear action, and the subject must update the response on the basis of constantly changing stimuli [10, 60]. Under these conditions, a stored cognitive map may be updated in agreement with new circumstances, or different internal cognitive maps may compete to generate the correct response [10], a feature that requires behavioral flexibility [8]. Hence, given that “new memories consist invariably of the updating and expansion of old ones which new experience activates by association and recall” [9], the cognitive map is also a dynamic map that is constantly updated in agreement with new perceptual information.

### 2.3. Chronic Stress Impairs PFC-Dependent Behavioral Tasks

It has been suggested that the maladaptive organization of behavioral responses observed in chronic-stress psychiatric disorders is related to the aberrant cognitive function of the PFC [61]. Chronic stress alters several executive functions of the PFC in humans, such as working memory, selective attention, and behavioral flexibility [62–64]. Similarly, chronic stress also affects executive functions mediated by the mPFC in rodents, like working memory [65–67], behavioral flexibility [62, 65, 68], recall of the extinction of conditioned fear [69–72], and decision making [73]. Hence, we suggest that these behavioral alterations have repercussions in the formation of cognitive maps for the perception-action cycle in the PFC, resulting in maladaptive behavioral responses, as observed in chronic stress-related psychiatric diseases.

## 3. Encoding and Representation of Cognitive Maps in the PFC

### 3.1. Neural Assemblies as Representation in the PFC

How are executive functions encoded in the PFC? A large body of evidence has shown that cognitive events take the form of modulations of the neuronal firing rate in the PFC. An early observation of this phenomenon was the modulation of the firing rate in the primate PFC that correlated to delayed response in working memory tasks [74–76]. Similarly to primates, changes in the firing rate in the rodent mPFC have been associated with different behavioral requirements [77]. Likewise, changes in firing rate in the mPFC have been associated with fear conditioning [78], motivational salience of places [79], control of fear expression [80, 81] strategy switches [82], recognition of safe versus unsafe places [83], object-in-place [84], and appetitive behavior [85].

However, due to variability of firing rates and limited representation capacity [86], the single-neuron spiking model is limited for encoding complex parameters [87]. Hebb [88] hypothesized that a discrete, strongly interconnected group of active neurons, the “neural assembly,” codes for distinct cognitive entities. The neural assembly hypothesis is based on the following three principles (reviewed extensively in [89]):

1. *The neural assembly is made up of a relatively small set of neurons that encode a behavioral parameter.* This principle assumes that a given representation is encoded by the synchronized activation of a population of neurons. The activation of the assembly is manifested by the synchronized firing of neurons, which is supported by synaptic connectivity among neurons that form the assembly. This principle also assumes that any given neuron is a member of different neural assemblies.
2. *As a behavioral parameter is learned, the neural assembly that represents it is formed.* This principle assumes that as a behavioral parameter is learned, the connectivity of the neurons that represent this behavioral parameter is gradually formed and strengthened through changes in synaptic weight (synaptic plasticity). It also assumes that the neural assembly is formed by the activity-dependent repeated coactivation of a group of neurons during behavior.
3. *Activation of the neural assembly correlates to behavior.* This assumes that behavioral performance is paralleled with activation of the neural assembly, thus providing a functional meaning to the assembly.

Therefore, a neural assembly that represents a cognitive map in the PFC is composed of functional connectivity among neurons whose coordinated activity reflects learned relationships with cognitive-relevant elements, such as strategies, decisions, and goals [90]. Experimentally, an assembly is a task-related synchronized overlapping firing of multiple neurons, and the task-dependent dynamics of the functional connectivity among multiple neurons [90]. Therefore, in order to detect neural assemblies it is necessary to record a large number of active neurons simultaneously during the execution of behavioral tasks [91]. In recent decades, Hebb's hypothesis of neural assemblies has been empirically supported thanks to advances in multielectrode recording techniques [91, 92] and statistical analysis for signal processing [87]. However, given that multielectrode recording is a highly invasive technique, most recordings of neural assemblies have been performed in freely moving animals, especially rodents. To date, the best-known examples of neural assemblies are “place cells” in the rodent HPC [93, 94] and the “grid cells” in the entorhinal cortex [95], which are considered different levels of internal representations of the location of the animal in space [93, 95].

### 3.2. Cognitive Functions Are Encoded by Neural Assemblies in the PFC

The emergence of neuronal assemblies related to executive functions like working memory has been well demonstrated in the rodent mPFC [96–98]. The activity of these assemblies related to working memory increases progressively in parallel to behavioral performance [98–100], suggesting that learning is related to the gradual formation of the assembly. In the study of Fujisawa et al. [101] the neural assemblies showed an increased firing specifically at the choice point of the T-maze in a working memory task. Similarly, Benchenane et al. [98] and Fujisawa and Buzsáki [102] showed that the activation of the assembly in the mPFC also occurs at the choice point in the Y-maze after learning, and that activation of assemblies predicts the choice of reward, suggesting that the assembly encodes for strategy representation. Thus, assemblies are well correlated with behavioral outcomes, suggesting that this is a neural representation of cognitive activity implemented by the mPFC. There is also evidence of neural assemblies in the mPFC relative to a set-shifting paradigm, a model of cognitive flexibility [103]. Importantly, these neuronal assemblies switch from encoding a familiar rule to a completely novel rule, and neural assemblies are predictive of behavioral choices even before a trial starts [103]. Altogether, the evidence presented suggests that neural assemblies support cognitive maps of the perception-action cycle, and that these neural assemblies are gradually formed in the mPFC during acquisition of a perception-action cycle.

The rodent mPFC is subdivided into PL and IL, and it has been suggested that both subdivisions have particular behavioral roles [9, 52, 53]. Some studies have not found significant differences in the neural representation of behavioral outcomes between these subdivisions [77–79, 99]. From these studies it could be suggested that there is no important difference between PL and IL in the representation of different aspects of goal-directed behavior. However, some studies reported important differences in the neural activity in PL and IL referent to behavioral correlates. The most well known differences between these structures are related to the control of the expression of conditioned fear. Milad and Quirk [104] found that neurons in the IL, but not PL, fire to the tone only when rats are recalling extinction of conditioned fear on the following day, which correlated to reduced freezing. On the other hand, Burgos-Robles et al. [80] found that neurons in PL fire correlated with fear expression, and that persistence of PL responses after extinction training was associated with failure to express extinction memory. These results suggest that PL and IL have opposite roles in the control of the expression of fear after extinction (reviewed in [105]). Differences between PL and IL have also been found in appetitive behavior. Burgos-Robles et al. [85] reported that PL neurons exhibited fast and transient responses to reward, whereas IL neurons exhibited delayed and prolonged activity to reward collection. Finally, Rich and Shapiro [82] found that neurons in PL and IL code for strategy switches; however, fire in the PL codes for anticipated adoption of new strategy, whereas IL neurons established new representation only after learning criteria have been established, suggesting that the two regions help to initiate and establish new strategies, respectively. Considering that PL and IL are strongly interconnected [106], altogether these results suggest that both PL and IL could represent different aspects of the same behavioral response.

### 3.3. Effect of Chronic Stress on Neural Assemblies

It has been suggested that dysfunction in neural assemblies is associated with stress-related disorders like SZ [107]. Indeed, there is evidence of impaired coordinated activation of firing in the mPFC in genetic and developmental models of schizophrenia [108, 109]. However, whether chronic stress impacts on neural assemblies that represent executive functions in the mPFC is unknown to date. Nonetheless, it has been shown that chronic stress disrupts stability of place cells in the HPC [110], impairment that is paralleled with decreased spatial memory. This suggests that chronic stress affects neural assemblies that code for cognitive functions in the PFC, likely altering the neural mechanism for their formation.

## 4. Mechanism for the Formation of Neural Representation of Cognitive Maps in the PFC

### 4.1. Role of Synaptic Plasticity in the Formation of Neural Assemblies

Hebb's hypothesis proposes that the formation, storage, and reactivation of neural assemblies depend on the alteration of the efficacy and connectivity of synapses in relevant neural networks [88], a concept known as synaptic plasticity [111]. This hypothesis poses that rapid, long-lasting change in synapse strength between consistently correlated pre- and postsynaptic activity drives strengthening of synaptic transmission, while weakly correlated activity drives weakening of synaptic transmission (“neurons that fire together, wire together”; [88]). Synaptic plasticity induced by neuronal activity, which generates neural network dynamics based on experience and training-induced activity, is especially important for the formation of assemblies with behavioral relevance [89]. Synaptic plasticity is implicated not only in the formation of neural assemblies, but also in their updating and long-term memory [112]. Long-term synaptic plasticity, the effects of which last from hours to days, plays an important role in the long-term storage of memory representations [112]. Experimentally, long-term synaptic plasticity has been assessed through long-term potentiation (LTP) and long-term depression (LTD), a long-lasting increase or decrease, respectively, in synaptic strength induced by a specific neural activity pattern, usually brief and strongly correlated to pre- and postsynaptic activity [113]. Importantly, synaptic plasticity results in structural remodeling of activated synapses, thus modifying structural connectivity among neurons (reviewed extensively by [114]). Thus, through long-term synaptic plasticity, sustained neural activity produces long-lasting synaptic modifications among neurons [115].

### 4.2. Synaptic Plasticity in the PFC

Short- and long-term synaptic plasticity have been observed in rodent mPFC slices* in vitro* [45, 116–123]. Also synaptic plasticity has been shown* in vivo* between the mPFC and other brain structures, as the HPC and the thalamus [124–128]. However, because of technical considerations, there is no direct evidence of the involvement of synaptic plasticity in the formation of neural assemblies in the mPFC. Some clues support the role of synaptic interactions in the formation of assemblies in the PFC. For example, Fujisawa et al. [101] found that 20% of the neurons of the assembly in the mPFC related to the choice of the animal in the T-maze showed monosynaptic interaction, suggesting that the efficacy of synaptic transmission between neurons varies according to task requirements. Courtin et al. [81] found that 63% of recorded neurons displayed excitatory or inhibitory interaction in the mPFC related to fear expression. These observations suggest that synaptic interaction is a critical process to integrate neurons in neural assemblies in the mPFC in relation to behavioral functions.

### 4.3. Chronic Stress Affects Synaptic Function in the PFC

It has been proposed that alteration in synaptic plasticity is an important mechanism in the development of stress-related psychiatric disorders [129–131]. Behavioral alterations induced by chronic stress are paralleled with alterations in synaptic transmission in the mPFC [72, 132], particularly in glutamatergic synaptic transmission [132, 133] (reviewed in [134]). The significance of these findings is that synaptic inputs from other brain systems, like the amygdala and HPC, as well as internal network connectivity, are mainly mediated by glutamatergic transmission [45, 46, 119]. It has been shown that glutamatergic synaptic transmission plays a critical role in firing activity in cortical neurons [135]. Indeed, chronic stress, either at the prenatal or at postnatal stage, reduces firing and burst activity of principal neurons in the mPFC* in vivo* [67, 71, 134, 136]. Chronic stress also reduces long-term synaptic plasticity in the mPFC both prefrontal in slices [133, 137] and* in vivo* in the HPC-mPFC axis [68, 138, 139], the mediodorsal thalamus-mPFC pathway [140], and the nucleus accumbens-mPFC neural pathway [141]. These data suggest that chronic stress impairs activity-dependent changes in synapse strength both in the internal circuitry of the mPFC and with the connectivity with other neural structures. These alterations are accompanied by severe disruption of working memory and cognitive flexibility [68, 140]. Interestingly, in both early life and adulthood, chronic stress blocked LTP of the hippocampal-mPFC pathway, impairment that was associated with an increased fear response after fear extinction [142, 143].

Whether chronic stress has direct repercussions on synaptic plasticity related to the formation of neural assemblies in the mPFC is still unknown. However, as Kim et al. [110] showed, the impaired formation of place cells induced by chronic stress in the HPC is accompanied by a decrease in LTP, suggesting that synaptic plasticity is an important mechanism for the stability of neural assemblies. The same principle can be applied to neural assemblies in the PFC.

### 4.4. Role of Neural Oscillations in the PFC

Neural oscillations are patterns of rhythmic electrical activity expressed as voltage changes in the extracellular space that arises from complex interactions between neurons and the networks to which they belong [144]. Given that long-term synaptic plasticity requires precise synchronization between pre- and postsynaptic neurons [145], it has been suggested that neural oscillations are responsible for synchronization of synaptic and neural activity involved in the formation and activation of neural assemblies [146–148]. This hypothesis, known as binding-by-synchronization [146], proposes that oscillations provide a “tag” that binds those neurons representing the same perceptual object. This is because oscillatory activity induces changes in the somatic membrane potential that strongly entrain action potentials, thus modulating both spike probability and timing [149, 150]. Thus, oscillatory activity may segregate the activity of neural populations that encode different aspects, or even segregate individual neural assemblies [89].

Oscillatory activity is significant because neuronal events related to cognitive activity are integrated by oscillatory rhythms [151]. For example, theta oscillations (4–10 Hz) arise during “active behaviors” and therefore synchronize specific neuron populations in the mPFC related to the requirements of the task, allowing the activation and emergence of neural assemblies [81, 98, 102, 108, 152–155]. Theta activity synchronizes spikes in the mPFC specifically in moments or places of special significance for the accomplishment of the task [98, 102]. Moreover, theta entrainment of prefrontal spikes, and not the firing rate, predicts successful working memory in rodents [154]. Benchenane et al. [98] showed that the activities of neural assemblies in the mPFC related to correct choice in a working memory task were synchronized to theta rhythm. Similarly, Fujisawa and Buzsáki [102] found that prefrontal goal-predicting neurons were significantly more synchronized by a 4 Hz oscillation than nonpredicting neurons. Moreover, synaptic transmission between pairs of neurons in the mPFC was significantly modulated by a 4 Hz rhythm in the choice point of the maze [102]. On the other hand, gamma oscillations (30–80 Hz), which emerge from local neural interactions, allow precise timing of action potentials by promoting the activation of a population of neurons and facilitating spike-dependent synaptic plasticity [147]. Theta oscillations entrain gamma activity in the mPFC, a modulation that is evident in the choice point in the T-maze and correlates with working memory performance [102, 156]. Therefore, through oscillatory synchrony, theta oscillations collectively coordinate gamma oscillations and neuronal assemblies in a task-relevant manner. Altogether, this evidence suggests that transient and dynamic synchronization of spikes to oscillatory activity in the mPFC is important to coordinate behavioral functions.

### 4.5. Oscillatory Spectral Coherence Synchronize Active Neurons in the mPFC

As described in Section 2.1, the efficient connectivity between the mPFC and other brain structures has a relevant role in the function of the control of behavior mediated by the mPFC. A special feature of neural oscillations is that they synchronize spikes and synaptic activity between different brain systems. Thus, spectral coherence, a measure of oscillatory synchrony between different brain areas [157], facilitates functional interactions and communication between neuron populations [151, 158], promoting synaptic plasticity for the formation of neural assemblies [159].

Oscillatory synchrony between the mPFC and HPC has been observed in rodents [102, 152, 153, 156, 160–166]. Interestingly, an increase in spectral coherence is observed in the theta range between the mPFC and the HPC during executive functions, such as working memory or inhibitory control [98, 102, 152, 153, 155, 156, 162]. This increase in spectral coherence at the theta frequency emerges in moments and places of special significance for the accomplishment of tasks [98, 102, 108, 153]. The increase in spectral coherence between the mPFC and HPC synchronizes mPFC spikes in a task-dependent manner, promoting the emergence and activation of neural assemblies that predict correct choice in a working memory task [98]. This work has important implications: first, to accomplish a given behavioral task, both the mPFC and HPC synchronize their oscillatory activity in the theta frequency, which is reflected as an increase in spectral coherence; second, an increase in coherence does not occur during the entire behavioral task, but only at relevant points in the maze; and finally, synchronization of oscillatory activity between the mPFC and HPC may provide, through spectral coherence in the theta frequency, the tag for neuronal communication of these structures required for the behavioral task.

### 4.6. Chronic Stress Affects Oscillatory Activity in the mPFC

Alterations in oscillatory activity have been suggested as a critical component in stress-related mental disorders [167–169]. Genetic and developmental models of SZ show reduced entrainment of prefrontal spikes to spectral coherence, suggesting a critical role of neuronal synchrony in physiopathology of psychiatric disorders [108, 109]. However, few studies have approached the effect of chronic stress on oscillatory activity in the mPFC. It has been shown that chronic stress decreases oscillatory synchrony between the thalamus and mPFC in the delta and theta frequency bands [139, 140]. Chronic stress also reduces spectral coherence between the mPFC and amygdala in the theta frequency band [170]. Oliveira et al. [171] showed that chronic stress-induced variations in HPC-to-mPFC coherence correlated with stress-induced behavioral deficits in a spatial reference memory task. Similarly, chronic stress increases spectral coherence at theta frequencies between the mPFC and HPC [172]. Therefore, we suggest that these chronic stress-induced alterations of oscillations impair synchrony between prefrontal neurons required for the activation of neural assemblies that represent executive functions.

## 5. Long-Term Storage of Cognitive Maps in the PFC

### 5.1. Memory Consolidation

Although not considered an executive function, the recall of remote memories has been shown to be linked to the PFC [173, 174]. It has been suggested that remote memory is associated with the long-term storage of cognitive maps of the perception-action cycle in the PFC [9, 56]. This static cognitive map in the PFC may link sparse representations stored in different brain systems, allowing bringing memories and other task-related knowledge “online” when needed and mixing new memories with existing ones [9, 56, 58]. Thus, this static cognitive map could be considered as a “schema” [175]. The transformation of the transient cognitive map of the perception-action cycle into a stable cognitive map occurs by a process known as memory consolidation, in which memories are gradually established from a relatively labile form (short-term or recent memory) to a more permanent state (long-term or remote memory) [176].

Currently, the most accepted model for memory consolidation is the two-stage model [177], which proposes that new information is first acquired and stored transiently during the waking state and then consolidated “off line” during sleep [178–181]. In this model, the interplay between the PFC and the HPC when subject is awake acts as a transient storage structure for recently acquired information [182, 183]. During slow-wave sleep (SWS), the interplay between the PFC and HPC may be required to consolidate the new cognitive maps in the PFC ([178] see below), inducing the increase and strengthening of neural connectivity within prefrontal networks [184]. Thus, the stable cognitive map is formed in the PFC and becomes independent of the HPC [173]. This process also allows the gradual integration of new memories with previously consolidated cognitive maps.

The proposed biological advantage of this two-stage model is that memories are not consolidated until the significance of an experience has been evaluated [185]. This implies that not all labile memories are transformed into remote memories. Indeed, it is well known that emotionally arousing experiences generally create strong, long-lasting memories [185], and that only memories that have motivational (or emotional) significance are consolidated [186, 187]. The amygdala, through the connection to the PFC, provides emotional information (both valence and arousal), whether aversive [188] or appetitive [189]. The activation of the amygdala by emotionally significant events, by tagging such events, facilitates the transmission of information between the HPC and the PFC needed for consolidation of cognitive maps (see below, [187]).

### 5.2. Oscillatory Interplay between the PFC and HPC Assists in the Long-Term Storage of Neural Assemblies in the mPFC

Neural assemblies that represent rules or strategies in goal-directed tasks are highly stable in the mPFC even for some time after acquisition, suggesting long-term stability of the neural assembly [103, 190]. It has been suggested that neural assemblies that represent cognitive events are consolidated through their reactivation during sleep. The first evidence of this phenomenon was the reactivation of place cells in the HPC during sleep [191]. In the mPFC, neuronal assemblies that are active and have behavioral relevance during wakefulness are replayed during SWS [96–98]. Interestingly, the replay of assemblies in the mPFC during SWS correlates with behavioral performance during wakefulness [96, 97, 103], suggesting that neural processes involved in the stabilization and consolidation of neural representation of cognitive activity carried out by neural assemblies take place during SWS.

It has been suggested that replay is accomplished by oscillatory synchrony between the neocortex and HPC [177]. During SWS, when delta activity is prominent [161, 163], particular patterns of spindle-shaped neural oscillations (periods of 1-2 s of waxing-and-waning patterns at 7–12 Hz, [192]) emerge in the neocortex, including the PFC, while sharp-wave ripples (SWRs, events of high-frequency 100–300 Hz oscillations, [193]) emerge exclusively in the HPC. Both SWR and spindles have been implicated in memory consolidation [192, 193]. During SWS, delta activity synchronizes prefrontal spindles and spikes to hippocampal SWR [160, 161, 163, 164]. Furthermore, putative pyramidal neurons and interneurons are modulated by spindles in the mPFC, showing enhanced responsiveness to hippocampal SPWRs during sleep [194]. This study also showed that gamma activity in the mPFC is modulated by spindles and SWRs, indicating that activity patterns in the mPFC and HPC are strongly synchronized by network oscillatory activity during SWS.

Synchrony between the mPFC and HPC also allows the replay of prefrontal neural assemblies during sleep [96–98]. Neural assemblies formed in the mPFC related to working memory are reactivated during sleep, especially in synchrony with hippocampal SWRs [97, 98]. This suggests that hippocampal SWRs serve to select neocortical neurons to be preferentially activated based on the information placed in hippocampal networks by past experience [160]. Together, these studies suggest that synchrony of discrete oscillatory patterns between the mPFC and HPC during SWS represents the offline transference of information from the HPC to cortical networks, a process that appears critical for the consolidation of memories. Thus, assembly recruitment by oscillatory activity during SWS seems to be critical for long-term memory consolidation of representations supported by these assemblies.

### 5.3. Possible Role of the Amygdala in the Consolidation of Cognitive Maps in the PFC

The amygdala promotes the transmission of information between the HPC and the neocortex needed for consolidation of memories by tagging events that are to be consolidated [187]. Interestingly, spectral coherence between the mPFC and the amygdala has been observed at the theta frequency [195, 196]. It has been proposed that oscillations in the amygdala, especially theta activity, are involved in the consolidation of emotionally significant memories [197, 198]. Theta activity is present in the amygdala only during emotional arousal [199], suggesting that this activity could inform the emotional value of current conditions. The amygdala has strong reciprocal connections with the mPFC and the HPC [46, 200] and theta activity between these structures is highly synchronous during emotional arousal [201–203]. Lesting et al. [195] showed that theta synchrony between the mPFC, HPC, and amygdala, as well as synchrony of neural firing, increased during retrieval of conditioned fear and decreased during extinction learning [195, 204]. Hence, it has been proposed that amygdalar theta activity during emotional arousal promotes memory consolidation by facilitating interactions between the mPFC and HPC [197]. Amygdalar theta oscillations could magnify the periods of effective synaptic interactions promoting synaptic plasticity in coactive structures like the mPFC and HPC during memory storage [197]. In support of this idea, an increase in spectral coherence at the theta frequency band during paradoxical sleep after fear learning between the mPFC, HPC, and amygdala has been reported, which correlated with behavioral performance [205]. Interestingly, about 10–20% of mPFC and amygdala neurons were activated and synchronized to theta activity during paradoxical sleep, suggesting the replay of neurons that represent fear learning [205]. Altogether, these results support the hypothesis that the amygdala, through oscillatory synchrony with the mPFC and HPC, provides a mechanism for interareal coordination related to aversive stimuli, providing contextual emotional information for subsequent behavior. Thus, communication through oscillatory synchrony may promote consolidation of emotionally significant memories.

### 5.4. Effect of Chronic Stress on Memory Consolidation

Some stress-related psychiatric disorders, like MDD and PTSD, are characterized by an aberrant persistence of aversive memories [3, 6]. Recent data indicate that chronic stress enhances retention of aversive long-term memories in rodents [206]. Also, chronic stress during gestation induces persistence of aversive long-term memory in the offspring at adulthood [136, 207]. This persistence of aversive long-term memory is paralleled with a decrease in the firing rate in the mPFC, and a reduced cross-correlation between hippocampal SWR and prefrontal spikes during delta activity [136], suggesting an association of synchrony of activity patterns between the mPFC and the HPC, and the persistence of aversive memories.

It is important to take into consideration that the aberrant persistence of aversive memories observed in stress related disorders is related to aversive memories [3, 6] but not to other types of memories. This suggests that chronic stress induces the selection of the memories to be consolidated, a function that may depend on the amygdala [187]. Other studies have reported that chronic stress generates hyperactivation of the amygdala [208]. For example, chronic stress increases firing in the amygdala [209] and also increases spectral coherence between the HPC and amygdala [172, 210]. Thus, the hyperactivation of the amygdala may strengthen memory consolidation for aversive experiences by increasing the number of neurons that best represent that event.

## 6. Summary: Effect of Chronic Stress

**Figure 2:**
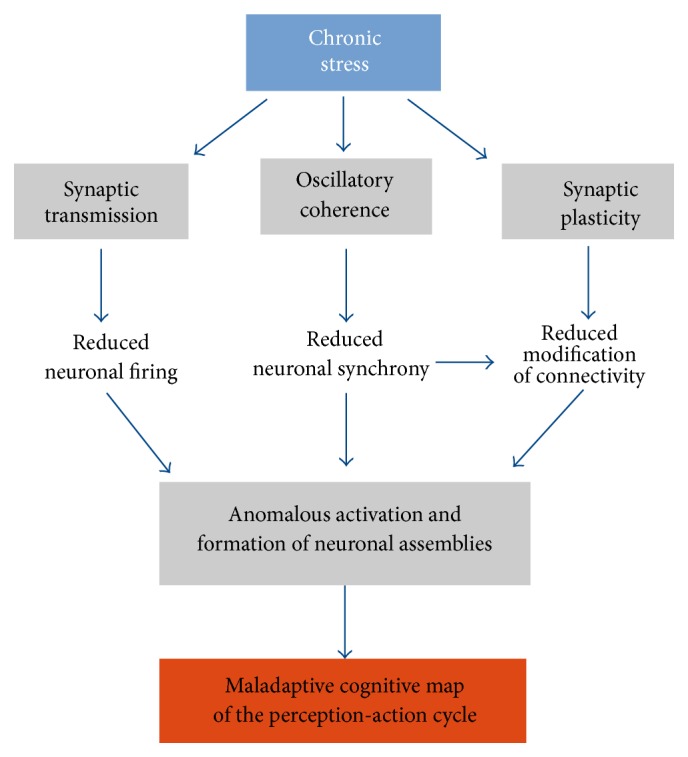
Proposed mechanism by which chronic stress affects the formation of neural assemblies related to cognitive maps in the PFC. In a first step, chronic stress reduces excitatory synaptic transmission in the PFC. This induces a reduction of firing in principal neurons. Chronic stress also reduces activity-dependent synaptic plasticity in the internal circuitry, and between the PFC and other brain systems. Also chronic stress reduces oscillatory coherence in cognitive relevant frequency bands between the PFC and other brain structures, resulting in a decreased synchrony between these and the PFC. The decreased oscillatory coherence together with the decreased synaptic plasticity results in an important reduction of the ability to modify functional connectivity dependent on neural activity in the PFC. Collectively, these impairments induce an aberrant formation and activation of neural assemblies in the PFC, which result in the development of maladaptive cognitive maps that link perceptual information to a repertory of erroneous actions, resulting in the maladaptive behavioral response directed to goals.

In the previous sections we integrate evidence from basic and cognitive neuroscience that approach how the mPFC participates in the implementation of the adaptive organization of behavioral responses. We also put evidence with focus on how chronic stress could affect the organization of behavioral responses mediated by the mPFC. This evidence, systematized in Figure 2, can be summarized as follows:

1. Chronic stress impairs glutamatergic synaptic transmission (Section 4.3), reducing the probability to firing of prefrontal neurons. Chronic stress also impairs long-term synaptic plasticity (Section 4.3), resulting in the impaired ability to modify synaptic connectivity in an activity-dependent manner between prefrontal neurons and between prefrontal neurons with neurons of other brain systems.
2. Chronic stress impairs also oscillatory coherence between the mPFC and other brain systems (as HPC and thalamus) in behaviorally relevant frequency bands (theta, gamma, and SWR) (Section 4.6). This impairs neural synchrony necessary for synaptic plasticity, impacting the formation of neural assemblies. On the other side, reduced neural synchrony affects the coordinated firing of prefrontal neurons, resulting in a reduced activation of previously formed neural assemblies.
3. Altogether, these alteration induced by chronic stress may result in the impairment of the formation and consolidation of neural assemblies that represent adaptive cognitive maps in the mPFC, in a similar manner as what occurs with hippocampal place cells ([110]; Section 3.3).
4. In both humans and rodent models, these neurophysiological alterations induced by chronic stress result in the impairment of the generation of behavioral responses dependent on the prefrontal network, such as working memory, selective attention, behavioral flexibility, recall of the extinction of conditioned fear, and decision making (Section 2.3). These behavioral alterations can be attributed to an inaccurate formation, consolidation, and activation of neural assemblies that represent cognitive maps in the PFC.

## 7. Hypothesis

The goal of the present review is to propose a framework that unifies cognitive neuroscience and neurophysiological evidence that attempts to explain the impairment of behavioral responses observed in chronic stress-related mental disorders. As a first proposal, our hypothesis is that the behavioral alterations observed in chronic stress-related psychiatric disorders can be categorized into three classes [1]: (i) exacerbated formation and implementation of maladaptive behavioral responses, a feature known as negative-cognitive bias, which manifests itself as the tendency to misinterpret perceptual stimuli as aversive or threatening [61, 211], (ii) exacerbated long-term recall of maladaptive memories, as increased retention and recall of aversive memories [3, 6, 15], and (iii) behavioral perseverance, manifested as the inability to adaptively update behavioral responses to novel environmental conditions [8].

The second proposal states that the above-mentioned cognitive defects observed in psychiatric diseases are the result of the anomalous and maladaptive formation of cognitive maps that represent perception-action cycles in the PFC. And the third proposal is that these anomalous representations are the consequence of the chronic stress-induced impairment of the neural mechanisms involved in the formation and/or consolidation of neural assemblies that represent these cognitive maps in the PFC.

### 7.1. Exacerbated Formation and Implementation of Maladaptive Behavioral Responses

These impairments result in the development of aberrant cognitive maps that link perceptual information to a repertory of erroneous actions, resulting in the maladaptive behavioral response directed to goals. How are these aberrant cognitive maps formed? In a first step, during the integration of perceptual information by the mPFC, the hyperactivation of the amygdala induced by chronic stress [209] can excessively transmit aversive-related information to the PFC, through a chronic stress-induced enhancement of spectral coherence at the theta band, between the amygdala and the mPFC [172, 210]. This could form an association between “neutral” perceptual environmental information and “aversive” information transmitted by the amygdala, resulting in a labile cognitive map and an incorrect perception-action cycle. This association is encoded by neural assemblies in the PFC that engage sparse representations stored in other neural systems with the aversive information [56]. Simultaneously, chronic stress decreases synaptic transmission and neural firing in the PFC [72, 132, 133, 136, 137], which results in an impairment for the implementation of executive functions, like working memory, selective attention, and inhibitory control [67–69]. The latter is required to inhibit amygdalar function [212], which may favor the acquisition of maladaptive cognitive maps, resulting in the implementation of maladaptive associations between different rules and strategies to accomplish different goals.

### 7.2. Exacerbated Long-Term Recall of Maladaptive Memories

In a second step, the long-term storage of these maladaptive cognitive maps may also be favored by chronic stress. Again, hyperactivity of the amygdala may favor synchrony at the theta oscillatory band between the PFC and the HPC during sleep, required to consolidate already formed cognitive maps in the PFC by favoring the replay of existing neural assemblies [205]. The chronic stress-induced decrease in PFC firing favors consolidation through an increased correlation between spikes and hippocampal SWR [136], resulting in the replay and stabilization of maladaptive neural assemblies in the PFC [96, 97]. Thus, labile acquired cognitive maps become stable (and maladaptive). This process can also be applied to the consolidation of aversive memories, as observed in PTSD.

In a third step, chronic stress may enhance activation (i.e., recall) of these maladaptive cognitive maps. Upon the presentation of ambiguous perceptual information, chronically stressed subjects activate more consolidated cognitive maps that correspond to previously acquired and consolidated maladaptive maps. This may be the consequence of the activation of neural assemblies that represent previously linked distributed associative events related to the negative-biasing interpretation of environmental information observed in chronic stress-induced disorders.

### 7.3. Impairment in Cognitive Flexibility

Chronic stress impairs cognitive flexibility as observed in reversal learning and set-shifting tasks [62, 68], suggesting that chronic stress impairs the ability to update previously formed cognitive maps or the perception-action cycle in the mPFC. Thus, even when environmental conditions change, stereotyped and maladaptive behavioral responses are maintained, as is observed in the impairment of extinction of conditioned fear induced by chronic stress [69, 72]. Importantly, neural assemblies that represent rules in the mPFC are updated with the change of contingency [103], suggesting that the impairment of behavioral flexibility prevents updating neural assemblies. For example, Wilber et al. [71] showed that chronic stress blocks the decrease of the firing rate in the mPFC required for successful recall of extinction of conditioned fear memory [80]. The impairment of synaptic transmission and plasticity in the mPFC induced by chronic stress [68, 132, 133, 137] may in turn impair the accommodation of previously formed neural assemblies, resulting in persistence of maladaptive cognitive maps for the perception-action cycle.

## 8. Concluding Remarks

In this review, we integrate evidence from cognitive neuroscience and behavioral neurophysiology with current knowledge of chronic stress-induced impairment in behavioral and neuronal function in the PFC to formulate a hypothesis on cognitive dysfunctions observed in stress-related neuropsychiatric disorders. We hypothesize that chronic stress-induced impairment of neural processes required for structuring neuronal assemblies in the PFC plays an important role in the abnormal organization of behavior observed in chronic stress-induced neuropsychiatric disorders. However, our hypothesis, to date, is mainly sustained by indirect evidence like the effect of chronic stress on the mechanisms involved in configuring neuronal assemblies, rather than observation of the effect of chronic stress on neural assemblies* per se*. Further research will complement and support the current hypothesis. It is necessary to record neuronal assemblies in the mPFC while executing cognitive functions in animals subjected to chronic stress. Large-scale neuronal recording methods [213] and high-resolution optical imaging techniques to assess the precise spatiotemporal properties of neuronal assemblies [214], together with manipulation of neuronal assemblies [215, 216], will offer a comprehensive picture leading us to an understanding of the physiopathology of chronic stress-related psychiatric disorders. These experiments offer the opportunity not only to clarify the relationship between chronic stress and neuropsychiatric disorders, but also to associate the modification of neural representations with behavioral performance.
